# Vitamin D and Obesity

**DOI:** 10.3390/nu5030949

**Published:** 2013-03-20

**Authors:** Simon Vanlint

**Affiliations:** Discipline of General Practice, School of Population Health, University of Adelaide, Adelaide, South Australia, SA 5005, Australia; E-Mail: simon.vanlint@adelaide.edu.au; Tel.: +61-8-8313-4634; Fax: +61-8-8303-3511

**Keywords:** vitamin D, obesity, overweight, calcium

## Abstract

Obesity is a significant health problem world-wide, particularly in developed nations. Vitamin D deficiency is pandemic, and has been implicated in a wide variety of disease states. This paper seeks to examine the consistently reported relationship between obesity and low vitamin D concentrations, with reference to the possible underlying mechanisms. The possibility that vitamin D may assist in preventing or treating obesity is also examined, and recommendations for future research are made. There is a clear need for adequately-powered, prospective interventions which include baseline measurement of 25D concentrations and involve adequate doses of supplemental vitamin D. Until such studies have been reported, the role of vitamin D supplementation in obesity prevention remains uncertain.

## 1. Introduction

Obesity, defined by the World Health Organisation as a body mass index (BMI) of 30 kg/m^2^ or more, is pandemic, affecting at least five million Australians and substantial numbers in most developed nations [1]. If overweight (BMI 25–29.9) is included, then approximately 14 million Australians, and 70% of Americans aged over 60, are obese or overweight [2]. Older people who have excess body fat accumulation face increased risk for coronary heart disease, hypertension, metabolic syndrome, osteoarthritis, diabetes mellitus, and other co-morbidities [3,4,5,6]. It is imperative that modifiable risk factors for obesity be identified, particularly those which might be readily addressed.

There is a consistent association in the published literature between increasing BMI and lower serum 25-hydroxyvitamin D (25D) concentrations. Early, smaller studies [7,8] reported an association between obesity and low serum 25D concentrations, as well as high concentrations of parathyroid hormone (PTH) and 1,25-dihydroxyvitamin D (1,25D). However, further, larger studies [9,10] found obesity to be associated with lower 25D concentrations, high PTH concentrations and low 1,25D concentrations. It has also been reported that body fat content is inversely related to serum 25D concentration, and that this associations is stronger than those between 25D and BMI and body weight [11]. A bi-directional genetic study, which limits confounding, has suggested that higher BMI leads to lower 25D, with the effects of lower 25D on BMI likely to be small [12]. The association between reduced 25D concentrations and obesity is therefore well-established, although the mechanisms for the lower 25D concentrations are not fully described, and there is uncertainty as to what the health consequences of these lower concentrations might be. This paper attempts to summarise the current state of knowledge regarding the causes of these reduced 25D concentrations, as well as the possible effects of vitamin D supplementation on obesity. 

## 2. Possible Mechanisms for Lower 25D Concentrations in Obese Individuals

### 2.1. Lower Dietary Intake

Vitamin D intake has been reported as being lower in obese men, but not women, when compared to their non-obese counterparts [13]. Low calcium and D intake have also been associated with obesity in both men and women [14], but this association does not necessarily imply a causal relationship. 

### 2.2. Reduced Cutaneous Synthesis

#### 2.2.1. Altered Behaviour

It is possible that obese individuals expose less skin to the sun less often than non-obese individuals, resulting in reduced synthesis of vitamin D. BMI, % body fat and sunbathing have been shown to be related in a population-based sample [15], although another study [16] found no relationship in a study of individuals aged over 65 years. This latter finding may be explained by the known decline in cutaneous vitamin D synthetic capacity associated with age [17]. It has also been noted that obesity results in larger body surface area [18], and thus could be expected to increase cutaneous vitamin D synthesis. 

#### 2.2.2. Reduced Synthetic Capacity

Concentrations of cutaneous 7-dehydrocholesterol (the substrate converted by ultraviolet light into previtamin D) appear not to vary between obese and non-obese individuals [19]. The existence of sufficient synthetic capacity is implied by the reduced risk of low serum 25D concentrations associated with outdoor exercise in obese individuals [20].

### 2.3. Reduced Intestinal Absorption

Hypovitaminosis D is well-documented in those who have had bariatric or gastric bypass procedures, in which a malabsorptive state is deliberately induced [21,22], but there is no evidence that obesity itself results in reduced absorption of dietary vitamin D. Given that vitamin D is fat-soluble, and that calcium absorption has been shown to be increased in diets high in fats [23], it is unlikely that obesity affects vitamin D-calcium homeostasis through altered gut absorption. 

### 2.4. Altered Metabolism

#### 2.4.1. Reduced Activation and/or Increased Catabolism

1,25D acts to limit production of its precursor, 25D [24]. Because early studies suggested that 1,25D concentrations were elevated in obese individuals, it was thought that this may lower 25D levels. Given that further, larger studies have suggested that 1,25D concentrations tend to be lower in obese individuals, this feedback mechanism is unlikely to be relevant. Adipose tissue (AT) in obese women expresses the enzymes for both the formation of 25D and its active metabolite, 1,25D and for degradation of vitamin D [25]. Subcutaneous AT has also been found to have lower expression of one of the enzymes responsible for 25-hydroxylation of vitamin D (CYP2J2), as well as a tendency toward a decreased expression of the 1-α hydroxylase. These data suggest that both 25-hydroxylation and 1-α hydroxylation are impaired in obesity. *In vitro* studies have demonstrated that 1,25D inhibits adipogenesis and induces adipocyte apoptosis [26,27]. Under normal physiological conditions the serum 1,25D concentration is tightly regulated, yet there can be significant differences between 1,25D concentrations within different tissues owing to in situ production. These factors make interpretation of the clinical significance of *in vitro* studies very difficult. 

#### 2.4.2. Sequestration of 25D in Adipose Tissue

Radio-labelling shows that 80% of vitamin D administered to rats is deposited rapidly in AT, from which it is then released very slowly [28]. Liquid chromatography/mass spectroscopy has shown a positive relationship between vitamin D in AT and serum 25D, consistent with AT being a storage site for 25D but not specifically implying sequestration [29]. These laboratory findings are consistent with clinical studies in which equal ultraviolet irradiation and also equal oral doses of vitamin D resulted in a 57% lower increase in serum 25D concentrations in obese individuals compared non-obese [19]. 

### 2.5. A Much Simpler Explanation

Despite the hypotheses and findings outlined above, an elegant study of 686 community-dwelling individuals showed that a volumetric dilutional model accounted for essentially all the variability in serum 25D concentrations attributable to obesity. Even though the factors described above may be operative, they are effectively “captured” by body weight. Once serum 25D concentrations in obese individuals are adjusted for body size, there is no longer a difference between obese and non-obese individuals [30]. The authors concluded that in obese individuals, vitamin D dosing for the treatment of deficiency should be based upon body weight, and calculated that an input of 70–80 IU/kg/day would be expected to produce serum 25D concentrations in the 75–100 nmol/L range.

## 3. Does Vitamin D Status Affect Obesity?

### 3.1. Background

Nuclear and membrane vitamin D receptors (VDRs) have been demonstrated in adipocytes, suggesting that AT is responsive to vitamin D [31]. A variety of mechanisms by which vitamin D and/or calcium may influence adiposity and energy balance have been proposed [32], but interventional studies have thus far been inconclusive, at least in part due to methodological issues. 

1,25D has been shown to have an *in vitro* anti-inflammatory effect on adipocytes, but the same study failed to demonstrate any reduction in systemic inflammatory markers *in vivo* in participants receiving 7000 IU oral vitamin D daily over an unspecified period [33]. Without knowing the pre-treatment 25D concentrations of the participants or the duration of oral supplementation it is difficult to interpret these findings. Baseline 25D concentrations were examined in relation to prevalent and cumulative incident obesity in a study of 2460 adults. In addition to prevalent obesity, serum 25D concentrations below 50 nmol/L were significantly associated with new-onset obesity [34]. Although this does not prove a causative effect, it is highly suggestive and warrants further clinical trials. 

### 3.2. Co-Administered Vitamin D and Calcium

Vitamin D is critical for calcium metabolism, and it is difficult to separate the effects of vitamin D from those of calcium, and of the two given in combination. Changes in weight, visceral fat mass and visceral fat area were compared in obese college students assigned to either a calorie-restricted diet with or without calcium 600 mg plus vitamin D 125 IU daily for 12 weeks in an open-label study. There was no difference in weight change between the groups, although the supplementation group did exhibit significantly greater decreases in visceral fat mass and fat area [35]. This study was limited by the very modest vitamin D dose used and the lack of baseline 25D measurement. Another randomised, double-blind 16-week study of 171 overweight and obese adults found that the addition of 1050 mg calcium with 300 IU of vitamin D daily was associated with a significant reduction in visceral adiposity compared with placebo [36]. 25D concentrations were not measured. Secondary analysis of data from a population-based, double-blind, placebo-controlled, randomised trial of 1179 postmenopausal women designed to determine the effects of calcium and vitamin D on osteoporotic fractures concluded that calcium supplementation over a four year period had a beneficial effect on body composition, but with no additional effect of vitamin D in the presence of a high calcium intake [37]. 

### 3.3. Vitamin D Alone

Turning to vitamin D administered without calcium, one study randomised 77 overweight and obese women to receive either 1000 IU of vitamin D daily or a placebo. In both groups the mean baseline 25D concentration was below 50 nmol/L, indicating deficiency. Vitamin D supplementation caused a significant reduction in body fat mass compared with placebo, as well as a significant rise in 25D concentrations. However, neither body weight nor waist circumference changed significantly in either group [38]. Changes in 25D concentration and changes in fat mass were significantly inversely correlated. A randomised study of 445 overweight and obese adults to receive either 20,000 IU of vitamin D twice weekly (DD), 20,000 IU once weekly plus placebo once weekly (DP), or placebo twice weekly (PP) for 12 months. 25D concentrations rose significantly in both the DD and DP groups, but not the PP group. There was no difference in weight change between the three groups. However, mean 25D concentrations in all three groups were above 50 nmol/L at baseline [39], which substantially reduces the impact of these findings. The overall impression is that vitamin D with or without calcium appears not to have a definite effect on weight, but that it may affect fat mass and distribution. However, the evidence is not yet complete enough to be compelling, and further research is needed. 

### 3.4. Effects of Weight Loss on Vitamin D Concentration

There is also evidence that weight loss leads to increased 25D concentrations, which may in turn provide additional protection against chronic disease. Data from 383 overweight or obese women who participated in a 2-year clinical trial of a weight-loss program showed that those who did not lose weight at 24 months had an increase in serum 25D of 1.9 ng/mL (4.8 nmol/L). However, 25D increased by 2.7 ng/mL (6.8 nmol/L) for those who lost 5%–10% of baseline weight, and by 5.0 ng/mL (12.5 nmol/L) for those who lost >10% of baseline weight (P = 0.014) [40]. These findings suggest that weight loss is associated with increased serum 25D concentration in overweight or obese women. 49% of participants were deficient (25D below 20 ng/mL (50 nmol/L)) at baseline. By study end 36% of all participants were deficient, with 17% of those who achieved a normal BMI being deficient.

## 4. Conclusions

The association between reduced 25D concentrations and obesity is well established, and can be adequately accounted for by a volumetric, dilutional model. Correction of low 25D concentrations in obese individuals requires higher doses than those often advocated for the general population. 

There are plausible mechanisms and some *in vitro* evidence supporting a role for vitamin D in weight reduction, with the proviso that it may be difficult to determine which effects are due to vitamin D itself and which are mediated via calcium. Clinical trials have not been conclusive, at least in part due to variable quality of study design. Some studies showing no effect of vitamin D supplementation on weight included participants who were vitamin D replete, and may thus have shown that giving supplemental vitamin D to those who are replete has no additional effect. There is a clear need for adequately-powered, prospective interventions which include baseline measurement of 25D concentrations and involve adequate doses of supplemental vitamin D. Until such studies have been reported, the role of vitamin D supplementation in obesity prevention remains uncertain.
